# Local weather is associated with rates of online searches for musculoskeletal pain symptoms

**DOI:** 10.1371/journal.pone.0181266

**Published:** 2017-08-09

**Authors:** Scott Telfer, Nick Obradovich

**Affiliations:** 1 Department of Orthopaedics and Sports Medicine, University of Washington, Seattle, United States of America; 2 Belfer Center for Science and International Affairs, Kennedy School of Government, Harvard University, Cambridge, United States of America; 3 Media Lab, Massachusetts Institute of Technology, Cambridge, United States of America; New York City Department of Health and Mental Hygiene, UNITED STATES

## Abstract

Weather conditions are commonly believed to influence musculoskeletal pain, however the evidence for this is mixed. This study aimed to examine the relationship between local meteorological conditions and online search trends for terms related to knee pain, hip pain, and arthritis. Five years of relative online search volumes for these terms were obtained for the 50 most populous cities in the contiguous United States, along with corresponding local weather data for temperature, relative humidity, barometric pressure, and precipitation. Methods from the climate econometrics literature were used to assess the casual impact of these meteorological variables on the relative volumes of searches for pain. For temperatures between -5°C and 30°C, search volumes for hip pain increased by 12 index points, and knee pain increased by 18 index points. Precipitation had a negative effect on search volumes for these terms. At temperatures >30°C, search volumes for arthritis related pain decreased by 7 index points. These patterns were not seen for pain searches unrelated to the musculoskeletal system. In summary, selected local weather conditions are significantly associated with online search volumes for specific musculoskeletal pain symptoms. We believe the predominate driver for this to be the relative changes in physical activity levels associated with meteorological conditions.

## Introduction

It is a commonly held belief that a causal relationship exists between local weather conditions and the joint pain and stiffness associated with musculoskeletal disorders [1,2]. These previous studies have generally been limited in terms of the time period assessed [3,4], geographical scope [5,6], or have focused on seasonal–rather than precise meteorological—variation [7].

Recently, researchers have begun to explore the potential of online search behavior as a method to infer information about health trends at the population level, exploiting the fact that the internet has become one of the primary sources used by individuals seeking health information [8,9]. Non-traditional data sources including Google Trends, Wikipedia page view data and Twitter data have the potential to be a rich source of information for healthcare researchers on population and regional level trends [10–12]. The potential utility of these data in the area of musculoskeletal health research has been demonstrated, with search volume-based time series data from the Google Trends tool having being found to contain significant seasonality and long term trends for searches related to foot and ankle pain [13].

If a relationship between relative search volumes for arthritis and general joint pain related terms and localized weather conditions exists, it may provide indirect evidence of the purported causal effect of weather conditions on these musculoskeletal issues at a population level, based on the assumption that elevated symptoms would drive an increase in information seeking behavior related to the condition and symptoms. Therefore, in this study, an analysis of localized online search volume for terms related to musculoskeletal joint pain and arthritis and their relationship to corresponding meteorological variables was explored.

## Materials and methods

Institutional Review Board approval was not required for this study as it did not involve collecting data directly from human participants. Search strategy reporting has been based on the checklist developed by Nuti et al. [14]. The top 50 cities in the United States by population were identified (based on 2014 estimates from the US Census Bureau) and the Google Trends web interface was used to obtain weekly relative search volumes for terms related to knee pain and stiffness (KNEE PAIN: “knee pain + painful knee + sore knee + stiff knee + knee stiffness”), hip pain and stiffness (HIP PAIN: “hip pain + painful hip + sore hip + stiff hip + hip stiffness”), and arthritis (ARTHRITIS: “arthritis + arthritic”) between 2011/01/02 and 2015/12/26 for these municipalities. In addition, weekly relative search data for one further search term (STOMACH PAIN: “stomach pain + painful stomach”) was obtained, this was designed to be a control term for pain searches unrelated to musculoskeletal problems. Note that in the Google trends tool, the “+” symbol in search terms represents the Boolean notation “OR”. The knee and hip were chosen as they are sites with a high prevalence of pain and injury [15,16], and pain and stiffness terms were used as these are often the primary symptoms of musculoskeletal disease or injury [17,18]. It is important to note that the Google Trends tool only provides relative data, normalized to a value of 100 index points which represents the peak search volume during the period of interest, therefore it is not possible to determine the exact number of searches being carried out. There were significant changes made to the geographical boundary definitions used by Google Trends on the start date used in this study, therefore trend data prior to this may not be directly comparable. Search terms were in the English language only, the all query category was used, and searches were carried out on 2016/03/01.

Corresponding daily summaries of historical local weather data from January 1st 2011 to December 31st 2015 for each city were obtained from the closest weather station that monitored temperature, relative humidity, barometric pressure, and precipitation. Complete details of the station locations are included in S1 Table. These weather variables were chosen as changes in these have previously been suggested to be associated with increases in musculoskeletal pain symptoms [3,5,19,20].

### Data processing

All analyses were carried out in R version 3.3.3. Complete datasets and analysis code are available at https://github.com/Telfer/GTrends_Arthritis_2016/.

For the search term data, city time series that were incomplete or displayed insufficient volume to provide a continuous series across the period of interest were not included in the analysis, nor were those that did not have a unique geographical region definition in the Google Trends tool. The weather variables maximum temperature, minimum temperature, average relative humidity, average barometric pressure, and sumtotal precipitation were extracted as daily time series from the weather datasets for each city. Cities with data that were incomplete for the period studied were not included in the analysis. To synchronize the weather data with the internet search volume data, each daily time series was converted to weekly time series data by averaging or summing over the relevant seven-day period.

Our theoretical relationship of interest is the effect of meteorological conditions on pain-related search activity of US residents. We empirically model this relationship as:

$$Y_{ist}=f({temp}_{ist})+X\zeta +\gamma_{t}+\nu_{is}+\epsilon_{ist}$$ (1)

In this time-series cross-sectional model fitted via ordinary least squares, *i* indexes cities, *t* indexes calendar weeks, and *s* indexes years. Our dependent variable *Y*_*ist*_ is an index that represents the relative search activity on the specific topic in city *i* on calendar week *t* in year *s*. Our main independent variable, *temp*_*ist*_, represents the weekly average of daily maximum temperatures over the contemporaneous week. Our relationship of interest is represented by *f*(*temp*_*ist*_), which provides separate indicator variables for each 5°C weekly average maximum temperature bin, allowing for flexible estimation of a nonlinear relationship between temperature and search activity [21,22]. We omit the 25–30°C indicator variable, and thus interpret our estimates as the change in search activity associated with a particular temperature range relative to the 25–30°C baseline for each of our search pain topics.

Further, the **X***ζ* term in Eq 1 represents an additional set of meteorological variables that include weekly sumtotal precipitation, average diurnal temperature range, average barometric pressure, and average relative humidity. We include these other meteorological variables as their exclusion might bias our estimates of the effect of included meteorological variables [23].

Unobserved characteristics may influence search activity in a particular city in a particular week. For example, people may exercise more–and thus search for exercise related pain more–in cities with better infrastructure or on days when they are more likely to have leisure time. To be sure that geographic and temporal factors like these do not bias our estimates, we include *γ*_*t*_, and *ν*_*is*_ in Eq 1. These terms represent calendar week and city-by-year indicator variables that account for unobserved characteristics constant across cities and weeks as well as temporal factors that might vary differentially by city [24]. Our identifying assumption, consistent with the literature [23,25,26], is that meteorological variables are as good as random after conditioning on these fixed effects. The estimated model coefficients can thus be interpreted as the effect of meteorological conditions on observed search activity [27,28].

## Results

**Fig 1:**
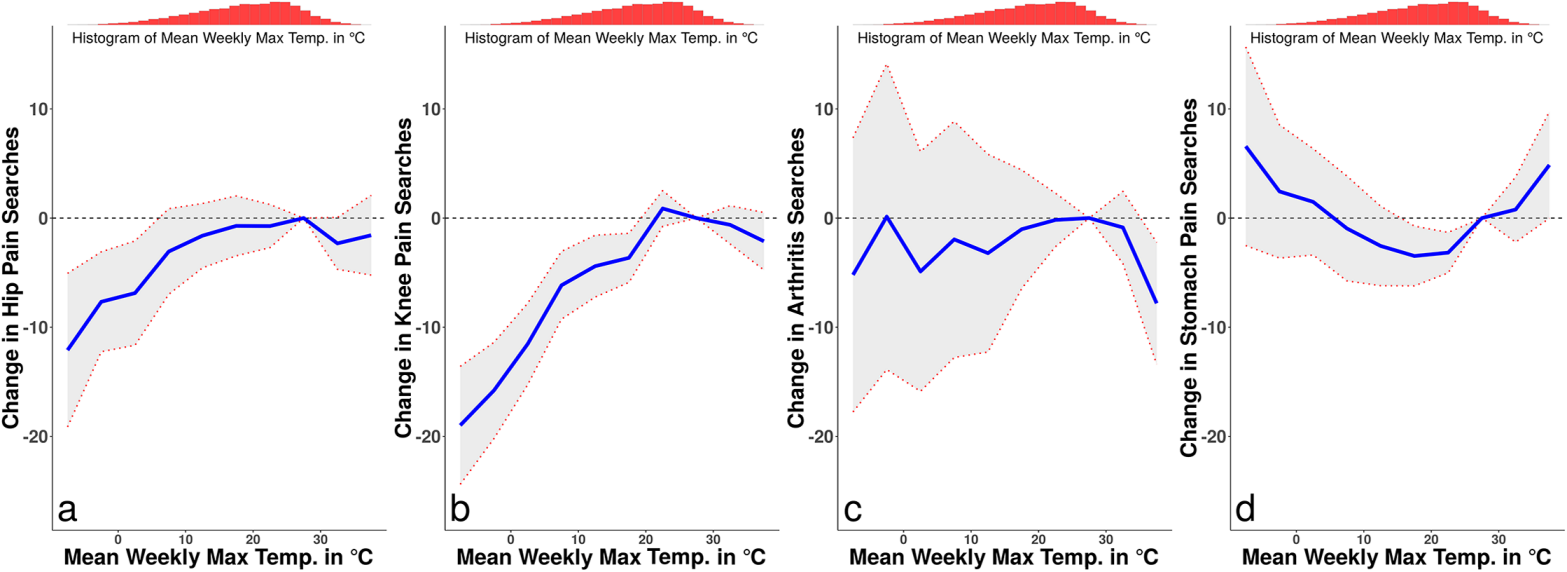
Maximum temperatures and searches for pain symptoms. This figure draws from the estimation of the model in Eq 1 on the weekly search behavior of citizens in 45 US cities between 2011 and 2015. It plots the estimated search activity associated with each maximum temperature bin for each search topic. Search activity for hip pain (panel (a)) and knee pain (panel (b)) increases up to 25–30°C (77-86F) and begins to decline past that point, though the effects of hotter temperatures are estimated with higher uncertainty. Search activity for arthritis symptoms (panel (c)) shows no significant effect of cold temperatures, but shows some decrease for markedly hot maximum temperatures. Search activity for our measure of non-musculoskeletal pain, stomach symptoms (panel (d)), indicates an inverse relationship, with search activity increasing in both cold and hot temperatures relative to more mild temperatures. Shaded error bounds represent 95% confidence intervals.

Full results from the regression analysis are presented in Table 1. Fig 1 panel (a), which presents the estimates of *f*(*temp*_*ist*_) from Eq 1, indicates hip pain related search activity increases up to 25–30°C and decreases past 30°C, though effects at higher temperatures are estimated with greater error. Average maximum temperatures around 0°C produce a reduction of approximately index points in hip pain search activity as compared to the 25–30°C baseline (coefficient: -6.874, p: 0.005, n: 9,087). Panel (b) of Fig 1 estimates Eq 1 for knee pain related searches. Average maximum temperatures around 0°C produce a reduction of approximately 12 index points in knee pain search activity as compared to the 25–30°C baseline (coefficient: -11.53, p: <0.001, n: 10,387). Panel (c) of Fig 1 produces estimates for arthritis pain related searches. We observe no significant effects of cold temperatures on arthritis search rates, though temperatures above 35°C produce a reduction of approximately 8 index points in arthritis pain search activity as compared to the 25–30°C baseline (coefficient: -7.812, p: 0.006, n: 11,687). Finally, panel (d) of Fig 1 suggests that the relationship between maximum temperatures and stomach pain related search activity is inverted from the musculoskeletal search activity. Cold temperatures and hot temperatures increase stomach pain related search activity relative to more mild temperatures.

**Table 1:** Regression table.

	Hip	Knee	Arthritis	Stomach
	(1)	(2)	(3)	(4)
tmax(-Inf,-5]	-12.100^***^	-18.991^***^	-5.205	6.579
	(3.589)	(2.756)	(6.407)	(4.641)
tmax(-5,0]	-7.668^***^	-15.788^***^	0.133	2.437
	(2.334)	(2.246)	(7.148)	(3.114)
tmax(0,5]	-6.874^***^	-11.530^***^	-4.894	1.481
	(2.437)	(1.903)	(5.600)	(2.482)
tmax(5,10]	-3.065	-6.160^***^	-1.956	-0.955
	(2.001)	(1.582)	(5.519)	(2.448)
tmax(10,15]	-1.613	-4.405^***^	-3.214	-2.559
	(1.504)	(1.445)	(4.620)	(1.857)
tmax(15,20]	-0.718	-3.644^***^	-1.022	-3.474^**^
	(1.399)	(1.147)	(2.770)	(1.396)
tmax(20,25]	-0.733	0.876	-0.171	-3.167^***^
	(1.008)	(0.835)	(1.250)	(0.946)
tmax(30,35]	-2.319^*^	-0.621	-0.863	0.782
	(1.211)	(0.893)	(1.685)	(1.524)
tmax(35, Inf]	-1.575	-2.125	-7.812^***^	4.865^*^
	(1.863)	(1.342)	(2.846)	(2.490)
Precipitation	-0.300^**^	-0.443^***^	-0.315	-0.042
	(0.134)	(0.106)	(0.197)	(0.107)
I(tmax—tmin)	-0.297	-0.003	-0.236	0.103
	(0.203)	(0.187)	(0.434)	(0.205)
Barometric pressure	-1.716	7.523^**^	9.445^*^	-5.582
	(3.521)	(3.312)	(5.468)	(4.891)
Relative humidity	-0.061	0.051	0.091	0.006
	(0.041)	(0.046)	(0.072)	(0.051)
Calendar Week FE	Yes	Yes	Yes	Yes
City:Year FE	Yes	Yes	Yes	Yes
*N*	9,087	10,387	11,687	6,749
R^2^	0.306	0.338	0.485	0.304
Adjusted R^2^	0.271	0.306	0.463	0.261
Residual Std. Error	21.864	19.805	33.058	22.737

Standard errors are in parentheses and are clustered on city and week. tmax: maximum temperature; press: tmin: minimum temperature.

***Significant at the 1 percent level

** Significant at the 5 percent level

* Significant at the 10 percent level

In addition to the effect of temperature on search activity, we observe a smaller, statistically significant linear effect of weekly precipitation (in centimeters) on both hip and knee pain related search activity (hip coefficient: -0.3, p: 0.025, n:9,087; knee coefficient: -0.443, p:, n:10,387).

## Discussion

This study analyzed online search volume data for musculoskeletal pain related terms in cities across the contiguous United States and found statistically significant associations with local temperatures and precipitation. To the authors’ knowledge this is the first study to use this type of methodology to investigate the relationship between local weather patterns and musculoskeletal pain symptoms. Although search volume data is a proxy measure of symptom prevalence, the 5-year analysis period here is considerably longer than most previous studies in this area, and covers a potential sample size of tens of millions, based on the population of the cities studied and adjusted for internet users and users of the Google search engine.

The most notable associations were found between increasing volumes of knee and hip pain searches and increasing temperatures (up to 25–30°C). These findings appear to be robust in terms of internet search data, as the search volumes for stomach pain, our control term, were found to have a very different pattern relative to temperature. Stomach pain information was more searched for during weeks where the temperature was either relatively low or high, and the minimum occurred between 15–25°C. As this type of pain is unlikely to be driven by the same mechanisms as musculoskeletal pain, the discordance between the search volume patterns provides confidence that the associations found between weather and searches for musculoskeletal pain are not simply an artifact of general internet usage during different weather conditions.

This analysis assumes that individuals with musculoskeletal problems will search for information about their condition and its symptoms during periods where those symptoms are elevated. Previous studies using online trend data to study healthcare related problems have shown good correlations between search data and safety alerts [12] and seasonality in prescribed pharmaceuticals [11]. While duplicate search terms from the same computer are not included in the analysis, it is possible that other search terms relating to the topics of interest could have been used. Search volume data is currently only available in weekly intervals for the period studied, somewhat limiting the temporal resolution of the analysis. Because search activity is geolocated to the city-level, measurement error may exist between the temperatures observed at a weather station and the temperature that an individual actually experienced, possibly attenuating the magnitude of our estimates [29]. We do not have information about the individuals performing the searches. Sampling bias may be present due to age, income, or preferred internet search engine. For example, older adults have the greatest prevalence of arthritis symptoms, and are less likely to use the internet. However, these usage rates are increasing: in the USA in 2014 over half of those over 65 reported being regular users [30], and in some cases searches may be conducted by another individual on behalf of the person with the symptoms. Due to insufficient data, several of the top 50 cities monitored had to be left out of the analysis. The Google Trends tool only records search volumes if the number of searches is greater than a certain threshold, the level of which is unknown, meaning that at certain times the data were missing or only available in monthly detail. It is possible that further developments to the Google Trends Tool, leading to better geographical boundaries will improve this.

There have been previous reports of elevated temperatures leading to increased pain after orthopaedic trauma [31], however studies in patients with arthritis have found no such relationship [3], or in some cases have reported negative correlations [5]. The positive correlation seen between temperature and joint pain search volumes in the present study may relate to general increases in activity patterns during warmer temperatures, potentially leading to more overuse and acute injuries during these periods [32,33]. In addition, small decreases were seen for knee and hip pain searches when temperatures increased to over 30°C. We can hypothesize that this pattern may be explained by individuals becoming more active—and therefore more at risk of injury—as temperatures increase up to a certain level, until it becomes too hot to exercise comfortably. This proposed pattern of increasing activity levels until a certain temperature has been reached and thereafter a decline in activity levels is supported by recent findings [34]. In the present study, no clear pattern was seen for arthritis related search terms as the temperature increased to 25–30°C, however we did see a significant reduction in search volume as temperatures increased above 30°C, similar to that seen for the knee and hip pain terms. The driving mechanism for this may also be related to a reduction in activity, or it may be due to some other effect that requires further investigation.

Other weather variables were found to be associated with search volumes. Most notable among these was precipitation, which was associated with a small but significant reduction in hip and knee pain search volumes. This again we hypothesize to be driven by a reduction in activity levels during periods of rain [34]. A significant effect of barometric pressure on knee pain searches was found. Increases in pain have been found to be associated with low barometric pressures after orthopaedic trauma [31], however in people with arthritis the opposite effect tended to be shown [6,19]. In contrast to barometric pressure and temperature, relative humidity was not found to be associated with any of the search term volumes. This weather variable has mixed evidence in for an effect on people with arthritis, with several studies finding an association [5,19,35], but others finding no effect [4,6,20]. Additional weather variables such as dew point may be more reflective of our perception of comfort than humidity [36], and could be the subject of further research to investigate this finding.

Future work will explore the relationship between internet search data and musculoskeletal pain further and may have implications for healthcare provision. These searches are rarely preceded by consultation with a medical professional, particularly in younger individuals [9], therefore, if significant and clinically relevant effects are reflected in the individual-level data, this information may allow healthcare providers to allocate resources more efficiently or provide different treatment strategies during time periods when temperatures are elevated, or by developing a surveillance tool using a model based on the search volume data and other factors. Online search data however requires careful assessment and use [37]. Perhaps the highest profile use of this type of data was in the case of Google Flu Trends [10], which showed initial promise as a surveillance tool that was able to detect regional influenza outbreaks faster than traditional disease monitoring techniques. However, reductions were subsequently found in the prospective accuracy of the tool and although modifications to the original tool have again improved its accuracy by, among other amendments, regularly updating the model using Center for Disease Control data [38], this case demonstrates the care that has to be taken with this type of data. Bearing in mind these caveats, these novel data sources do however remain an active area research, with many groups around the world applying it to different healthcare related problems [39–42].

A model to predict the prevalence of musculoskeletal pain would require significant further development of the methodology and validation against individual level data to determine if changes in search volumes are indeed related to changes in individual specific pain and healthcare seeking behavior. External factors such as economic conditions have been shown to be associated with increases in musculoskeletal search volumes and may need to be included in any predictive model [43]. In addition, future work may supplement these analyses by studying search terms in different languages, and across different regions to determine if these results are consistent on a global scale.

This study provides further confirmation that the study of online search data may have the potential to provide insights into healthcare related issues at the population level. Further work is required to determine if a local surveillance tool for musculoskeletal conditions can be developed based on the search volume data.

## Supporting information

S1 Table Weather station locations. (DOCX)
