# Supplementary material for: Local weather is associated with rates of online searches for musculoskeletal pain symptoms
Source: PLoS One. 2017 Aug 9;12(8):e0181266. doi: 10.1371/journal.pone.0181266 (PMC5549896; doi:10.1371/journal.pone.0181266)
Supplement: S1 Table — (DOCX) [file pone.0181266.s001.docx]

**S1 Table**

| **CITY** | **STATE** | **STATION** | **WEATHER DATA** | **TREND DATA** |
| --- | --- | --- | --- | --- |
| NEW YORK | NEW YORK | CENTRAL PARK | Yes | Yes |
| LOS ANGELES | CALIFORNIA | LOS ANGELES DOWNTOWN | Yes | Yes |
| CHICAGO | ILLINOIS | CHICAGO MIDWAY | Yes | Yes |
| HOUSTON | TEXAS | HOBBY | Yes | Yes |
| PHILADELPHIA | PENNSYLVANIA | NORTHEAST PHILADELPHIA | Yes | Yes |
| PHOENIX | ARIZONA | PHOENIX SKY HARBOUR INTERNATIONAL | Yes | Yes |
| SAN ANTONIO | TEXAS | SAN ANTONIO INTERNATIONAL | Yes | Yes |
| SAN DIEGO | CALIFORNIA | SAN DIEGO INTERNATIONAL LINDBERGH | Yes | Yes |
| DALLAS | TEXAS | DALLAS LOVE | Yes | Yes |
| AUSTIN | TEXAS | AUSTIN | Yes | Yes |
| JACKSONVILLE | FLORIDA | JACKSONVILL NAVAL AIR STATION | Yes | Yes |
| SAN FRANCISCO | CALIFORNIA | SAN FRANCISCO INTERNATIONAL | Yes | Yes |
| INDIANAPOLIS | INDIANA | INDIANAPOLIS INTERNATIONAL | Yes | Yes |
| COLUMBUS | OHIO | OHIO STATE UNIVERSITY | Yes | Yes |
| CHARLOTTE | NORTH CAROLINA | CHARLOTTE DOUGLAS INTERNATIONAL | Yes | Yes |
| DETROIT | MICHIGAN | DETROIT CITY | Yes | Yes |
| SEATTLE | WASHINGTON | BOEING FIELD KING COUNTY INTERNATIONAL | Yes | Yes |
| DENVER | COLORADO | CENTENNIAL | Yes | Yes |
| WASHINGTON | DC | RONALD REAGAN WASHINGTON INTERNATIONAL | Yes | Yes |
| MEMPHIS | TENNESSEE | MEMPHIS INTERNATIONAL | Yes | Yes |
| BOSTON | MASSACHUSETTS | LOGAN INTERNATIONAL | Yes | Yes |
| NASHVILLE | TENNESSEE | NASHVILLE INTERNATIONAL | Yes | Yes |
| BALTIMORE | MARYLAND | BALTIMORE-WASHINGTON INTERNATIONAL | Yes | Yes |
| OKLAHOMA CITY | OKLAHOMA | WILEY POST | Yes | Yes |
| PORTLAND | OREGON | PORTLAND INTERNATIONAL | Yes | Yes |
| LAS VEGAS | NEVADA | MCCARRAN INTERNATIONAL | Yes | Yes |
| LOUISVILLE | KENTUCKY | LOUISVILLE INTERNATIONAL STANDIFORD | Yes | Yes |
| MILWAUKEE | WISCONSIN | GEN MITCHELL INTERNATIONAL | Yes | Yes |
| ALBUQUERQUE | NEW MEXICO | ALBUQUERQUE INTERNATIONAL SUNPORT | Yes | Yes |
| TUSCON | ARIZONA | DAVIS MONTHAN AIR FORCE BASE | Yes | Yes |
| FRESNO | CALIFORNIA | FRESNO YOSEMITE INTERNATIONAL | Yes | Yes |
| SACRAMENTO | CALIFORNIA | SACRAMENTO EXECUTIVE | Yes | Yes |
| KANSAS CITY | MISSOURI | KANSAS CITY INTERNATIONAL | Yes | Yes |
| ATLANTA | GEORGIA | DEKALB PEACHTREE | Yes | Yes |
| VIRGINIA BEACH | VIRGINIA | OCEANA NAVAL AIR STATION | Yes | Yes |
| OMAHA | NEBRASKA | EPPLEY AIRFIELD | Yes | Yes |
| RALEIGH | NORTH_CAROLINA | RALEIGH-DURHAM INTERNATIONAL | Yes | Yes |
| MIAMI | FLORIDA | MIAMA INTERNATIONAL | Yes | Yes |
| MINNEAPOLIS | MINNISOTTA | CRYSTAL | Yes | Yes |
| TULSA | OKLAHOMA | JONES JR | Yes | Yes |
| CLEVELAND | OHIO | BURKE LAKEFRONT | Yes | Yes |
| WICHITA | KANSAS | WICHITA EISENHOWER NATIONAL AIRPORT | Yes | Yes |
| NEW ORLEANS | LOUISIANA | NEW ORLEANS INTERNATIONAL | Yes | Yes |
